# Evaluating the Effectiveness of Smart Glasses in Reducing Patient Care Time in Emergency Departments: Cohort Study From the Hangzhou Asian Games

**DOI:** 10.2196/65617

**Published:** 2025-06-30

**Authors:** Xinwei Jiang, Bangbo Xia, Mohammad Mostafa Ansari, Huiquan Jiang, Jianjiang Qi, Zhongheng Zhang, Sheng Dai, Pingping Zheng, Yang He, Ning Liu, Pengpeng Chen, Ronghua Luo, Xuchang Qin, Yansong Miao, Junru Dai, Xiaoyu Zhou, Changliang Wang, Hui Chen, Wenbin Xu, Tao Wu, Qiang Shi, Zhonghua Chen, Liping Zhou, Hao Zhang, Yun Xie, Quan Zhang, Bifa Zhou, Xiaohong Pan, Zixi Chen, Libo Zhen, Yaqing Sun, Zelin Lu, Yihao Loh, Shameera Sayer, Jennifer Mochtar, Pannika Wongpraewit, Yifan Wang, Yucai Hong

**Affiliations:** 1 Nursing Department, Sir Run Run Shaw Hospital, Zhejiang University School of Medicine, Hangzhou, China; 2 Department of Emergency Medicine, Sir Run Run Shaw Hospital, Zhejiang University School of Medicine, Hangzhou, China; 3 School of Medicine, Zhejiang University, Road Qingchundong No. 3, Shangcheng District, Hangzhou, 310016, China, 86 87951669, 86 87951669; 4 Medical Department, Organising Committe, Hangzhou Asian Games, Hangzhou, China; 5 Hangzhou Seventh People’s Hospital, Hangzhou, China; 6 Department of Medical Affairs, Sir Run Run Shaw Hospital, Zhejiang University School of Medicine, Hangzhou, China; 7 Department of Emergency Medicine, Hangzhou First People's Hospital, Hangzhou, China; 8 Hangzhou Red Cross Hospital, Hangzhou, China; 9 Department of Intensive Care Medicine, Sir Run Run Shaw Hospital, Zhejiang University School of Medicine, Hangzhou, China; 10 Department of Cardiology, Sir Run Run Shaw Hospital, Zhejiang University School of Medicine, Hangzhou, China; 11 Department of Orthopaedics, Sir Run Run Shaw Hospital, Zhejiang University School of Medicine, Hangzhou, China; 12 Department of Rehabilitation Medicine, Sir Run Run Shaw Hospital, Zhejiang University School of Medicine, Hangzhou, China; 13 Department of General Surgery, Sir Run Run Shaw Hospital, Zhejiang University School of Medicine, Hangzhou, China

**Keywords:** telemedicine, teleconsultation systems, emergency medicine, emergency care, patient care, multidisciplinary treatment, treatment, efficiency, smart glasses, augmented reality, wearable technology, wearable devices, EHR, electronic health record, retrospective study, effectiveness, Chinese population, Asia, Asian Games

## Abstract

**Background:**

Challenges in emergency medicine include overcrowding, insufficient emergency care resources, and extended emergency department (ED) waiting times. These issues contribute to delays in treatment and unfavorable outcomes. This situation worsens in events with large crowds and particularly worsened during the COVID-19 pandemic. The integration of augmented reality (AR) smart glasses could potentially enhance patient care in the ED.

**Objective:**

This study aims to assess the effectiveness of AR smart glasses in reducing patient care time in the ED during the 19th Asian Games and the Fourth Asian Para Games Hangzhou 2022 (HAG2022). The study specifically compares the prepreparation time (PPT) and consult response time (CRT) in patients receiving teleconsultations via AR smart glasses versus those receiving standard care without AR.

**Methods:**

This retrospective study was conducted between September 13, 2023, and October 28, 2023, during HAG2022. The data were gathered from AR smart glasses using 5G technology at the HAG2022 village and electronic health records at Sir Run Run Shaw Hospital, China. The study included 2 groups: the teleconsultation by augmented reality telemedicine system (ARTS) group and the non-ARTS group. The main data assessed were PPT and CRT in ED.

**Results:**

During the research period, 80 patients were divided into 2 cohorts: the ARTS cohort (n=10) and the non-ARTS cohort (n=70). Gender and age demographics showed no significant differences between the cohorts. The ARTS cohort had a significantly lower average PPT of 23 minutes compared to the non-ARTS cohort’s 40.3 minutes (*P*<.001). In addition, CRT in the ARTS cohort was significantly lower at 15.6 minutes compared to the non-ARTS cohort’s 164.8 minutes (*P*=.03). The outcomes suggest that smart glasses are effective in decreasing PPT and CRT.

**Conclusions:**

AR smart glasses have the potential to enhance patient admission efficiency and reduce care time in EDs. However, despite these benefits, further research is needed to confirm their effectiveness, and additional studies are essential to identify the challenges and barriers to their successful implementation in emergency medicine.

## Introduction

Emergency departments (EDs) play a pivotal role in providing immediate care for patients with acute illnesses or injuries [1]. EDs face several common problems that hinder their efficiency, including overcrowding and resource limitations. Overcrowding results in prolonged waiting times, length of stay, diminished quality of care, and increased mortality and morbidity rates [2-5]. Furthermore, requiring diagnostic tests and specialist consultations also contribute to extended waiting times and decision-making in EDs [6-8]. The time spent waiting for a specialist to arrive, conduct the consultation, and make a decision constitutes 33% of the length of stay for admitted patients and 54% for discharged patients in ED, highlighting a significant delay that contributes to longer waiting times [9]. Research has revealed that delays in ED care, such as those caused by overcrowding, can significantly impact patient outcomes, particularly for severe conditions like acute stroke [10,11]. A 2021 study reported an 8% increase in 30-day mortality for patients waiting more than 6‐8 hours in the ED [12].

The 19th Asian Games and Fourth Asian Para Games Hangzhou 2022 (HAG2022), held from September 13 to October 28, 2023, in Hangzhou, Zhejiang province, China, marked the largest sporting event in Asia, with more than 12,000 athletes. While most events occurred in Hangzhou, several other cities in Zhejiang province also hosted competitions [13]. The global pandemic led to the postponement of HAG2022 to 2023 [14], prompting the organizing committee to develop a detailed medical emergency plan to ensure participant safety [15]. Previous experiences have shown that hosting a large-scale event during and after the COVID-19 pandemic posed considerable challenges for medical systems [16]. Such extensive public gatherings impose significant demands on the local emergency medical services (EMS) [17,18]. Therefore early identification of patients most likely requiring consultation, and limiting unnecessary consultations is important to improve EDs services [19] [20].

EMS served as the first line of response during HAG2022. The process began when patients or others contacted the national emergency hotline (120). The medical call center then dispatched ambulances based on the incident’s location and urgency. After assessing the patient’s condition at the scene, they were either transferred to the polyclinic at the HAG2022 village or, if necessary, to designated hospitals outside the village for advanced care. During this process, crucial information, including the patient’s chief complaint, medical history, symptoms, and examination results, was collected and transmitted via 2-way radio to the hospital, allowing for immediate preparation and treatment upon the patient’s arrival [21].

Traditional telecommunication methods, such as radio transmissions from the HAG2022 village or an ambulance to the hospital, are time-consuming and hinder continuous patient monitoring. This delay can lead to slower diagnoses and worsening clinical conditions [22].

Telemedicine, enhanced by augmented reality (AR) technology, addresses various challenges by providing visual aids and facilitating specialist communication for remote patient evaluations in outpatient, inpatient, and emergency settings [23]. Smart glasses enhance collaboration for first responders by allowing them to perform triage and treatment without obstruction while enabling physicians in tertiary care to prepare for coming patients [23-25].

This research aims to evaluate the impact of using smart glasses on EDs preparedness and patient care during HAG2022. Key parameters under review include waiting time and time to get specialist consultations in emergency department. To our current knowledge, this research stands as one of the first investigations delving into using smart glasses in ED during a sports event.

## Methods

### Study Design

This retrospective study was conducted at Sir Run Run Shaw Hospital (SRRSH), China. This study aims to compare the outcomes of 2 groups: the intervention group, using the AR smart glasses, and the control group, adhering to the standard conventional triage process without the AR tool, only using a 2-way radio connection. All calls for emergency care within the HAG2022 village were directed to a central medical call center and subsequently triaged for ambulance transport. Randomization into the teleconsultation by augmented reality telemedicine system (ARTS) and non-ARTS groups was determined based on the availability of AR smart glasses for teleconsultation at the time of each case. Cases were not pre-randomized; instead, they were categorized retrospectively based on whether the ARTS was used.

### Augmented Reality Tool

**Figure 1.**
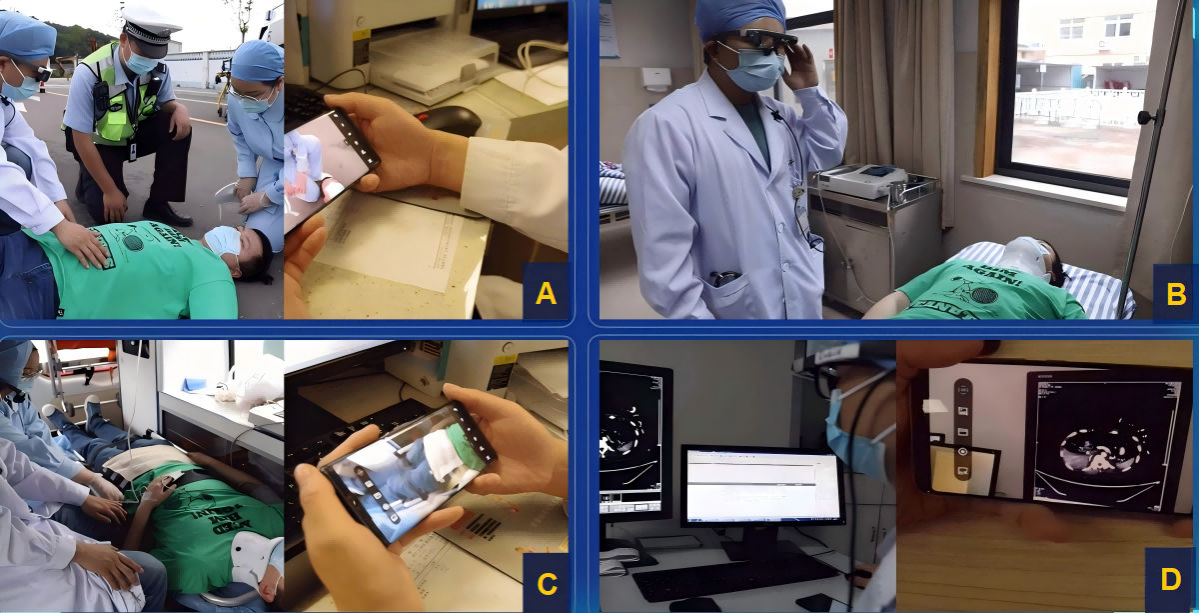
(A) On-site rescue; (B) management at primary medical stations; (C) comprehensive ambulance transportation; and (D) multidisciplinary teleconsultation at the tertiary hospital.

The AR tool used in this study was a smart glasses manufactured by Rokid AR Studio. It was Rokid glass 2 model released in 2020. The device operated on Android 9.0 and it connected to 5G connection with a bandwidth speed 30-mbps delivering a very low latency. The device supported real-time screen sharing and therefore the data were collected from smart glasses including visual and audio with real-time 2-way communications. Physicians at the hospital can access the information and video via a mobile app, enabling continuous monitoring of patients. A total of 10 AR smart glasses were deployed across various operational units during the study. These included 2 units in the Field of Play, 7 in hotels located outside the Asian Games Village, and one in medical polyclinic within the Asian Games Village. In the end, AR smart glasses were used for teleconsultation in only 1 hotel outside the Asian Games Village and 2 medical polyclinics within the village (see Figure 1). Among the medical polyclinics in the Asian Games Village, one was designated as a standard emergency room, equipped with advanced diagnostic tools such as CT imaging, bedside ultrasound, and basic blood testing capabilities. The remaining polyclinics were operated as general outpatient clinics, staffed with senior attending physicians specializing in general or emergency medicine, and supplied with commonly used medications. Each operational team was composed of trained paramedics, who were responsible for managing the AR smart glasses to enable telemedicine operations.

### Participants

The study, conducted between September 13, 2023, and October 28, 2023, included the recruitment of all patients (ages 16 years and older) presenting with acute illnesses who were admitted to the ED of SRRSH from the HAG2022 village polyclinic or the field of injury. These patients included athletes, attendees, and other individuals present in the HAG2022 village during the event. SRRSH served as the primary tertiary referral center for the HAG2022 medical plan, and while other medical facilities were available, this study exclusively focused on cases managed at SRRSH.

Exclusion criteria included cases with incomplete data, technical difficulties with AR smart glasses, or loss of internet connection. All eligible patients presenting to the SRRSH ED during the study period were assigned to either the ARTS (intervention) or non-ARTS (control) groups based on the use or non-use of AR smart glasses for teleconsultation.

### Outcomes

**Figure 2.**
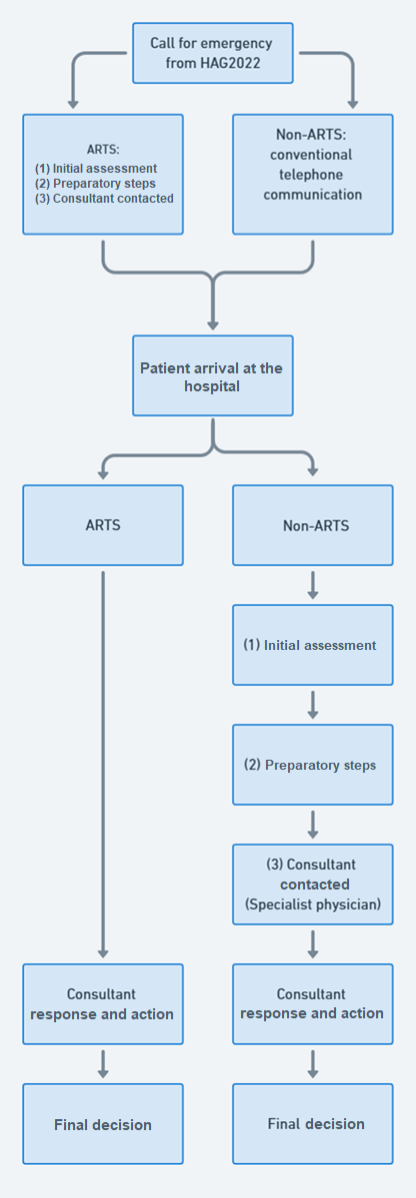
Comparison of patient admission workflows using the augmented reality telemedicine system (ARTS) versus conventional methods (non-ARTS). HAG2022: 19th Asian Games and Fourth Asian Para Games Hangzhou 2022.

The main outcomes assessed in this study were the prepreparation time (PPT) and the consult response time (CRT). PPT refers to the time interval from the patient’s arrival at the tertiary hospital to the completion of preparatory steps required for their subsequent management. These steps include the collection of necessary information, initial assessments, diagnostic tests, imaging, and stabilization measures before a decision is made on discharge, admission, or further treatment. For patients and athletes involved in the Asian Games, additional time may be required for registration compared to ordinary patients residing in China. Registration processes often took longer due to factors such as unfamiliarity with international names, particularly distinguishing between surnames and given names, and ensuring the accurate typing of full names, which was critical for medical insurance processing and certification. Furthermore, preparatory steps such as diagnostic tests and imaging often consumed additional time, depending on whether the necessary information was available before or after the patient’s arrival at the tertiary hospital. Pre-arrival collection of patient information had the potential to significantly reduce the waiting time by enabling faster ordering of diagnostic tests and imaging upon arrival. CRT is defined as the time from when the consulting physician is contacted to the moment they provide a response. CRT is critical for time-sensitive conditions such as acute coronary syndrome, stroke, trauma, and septic shock, where delays in consultation can negatively affect outcomes. Traditional consultation methods, like telephone or static video, often lack the ability to capture detailed clinical data. The use of AR glasses improved CRT by providing consultants with a first-person, real-time view of the patient’s condition, allowing for the assessment of critical parameters such as pupil response and gait analysis. AR technology enabled up to six consultants to participate simultaneously, using standard smartphones, regardless of location. This innovation can reduce CRT, enhance consultation quality, and demonstrate scalability, particularly in resource-constrained settings or during emergencies. To enhance clarity, a workflow diagram was added to illustrate the emergency care process and the measurement points for PPT and CRT (see Figure 2). The decision to focus on PPT and CRT as primary metrics stems from their direct reflection of triage efficiency and consultant responsiveness, which are critical in emergency medicine. While broader metrics such as length of stay, time to admission, or time to discharge are relevant, they are influenced by multiple systemic factors, including resource availability, hospital workflows, and patient-specific variables. PPT and CRT, on the other hand, specifically measure the timeliness of preconsultation and consultation processes, which are particularly important in optimizing emergency care during large-scale events like the Hangzhou Asian Games.

### Data Collection

There were specially experienced paramedics and physicians from both HAG2022 polyclinic and SRRSH who volunteered to participate in the study. They underwent thorough training and familiarization sessions to ensure proficient use of the AR tool before its deployment. Specialized teams of trained personnel were used, thereby enabling regular ED staff to continue their routine responsibilities without incurring additional workload. A structured data collection form was developed to record demographic information, triage times, and CRT using electronic health records. The patients were divided into the teleconsultation by the ARTS and the non-ARTS groups. Patients in the ARTS group received teleconsultation with smart glasses before they arrived at the SRRSH. The AR functionalities, including real-time visualization of patient data and interactive guidance, empowered the health care professionals at SRRSH to not only document basic information, chief complaints, and vital signs but also obtain additional information per scenario. Furthermore, they managed to establish a connection with the AR tool at any point during the transportation. Patients in the non-ARTS group underwent standard conventional triage procedures, where the hospital was notified of patient admission via telephone communication. Information such as the chief complaint, vital signs, and general condition was reported to the hospital by paramedics through telephone communication (see Multimedia Appendix 1). Patient privacy, confidentiality, and data protection were strictly maintained throughout the study by assigning numbers to each patient instead of using identification information.

### Statistical Analysis

The study’s sample size was calculated using the formula: sample size = (Zα/2+ Zβ)² * σ² / Δ², where Zα/2 is the critical value corresponding to the significance level, Zβ is the critical value for the study’s power, σ is the estimated SD of the population, and Δ is the minimum detectable effect size. SPSS Statistics software (version 20.0; IBM), was used for statistical analysis and data management in this study. Data analysis used the *χ*^2^ test, Welch *t* test, binary logistic regression analysis, and multiple linear regression analysis, to compare the PPT and CRT between the intervention and control groups.

### Ethical Considerations

Ethical approval for this study was obtained from the Research and Ethics Committee at Zhejiang University Affiliated Sir Run Run Shaw Hospital (registry 2023-850-01). Given the retrospective nature of the study and its use of anonymized clinical data, the study was granted an exemption from obtaining additional informed consent. The ethics committee granted a waiver of informed consent, as the study did not interfere with the regular triage procedures or prevent participants from receiving necessary medical treatment. All data were anonymized to ensure privacy and confidentiality, with identifying information replaced by numerical codes. The study adhered to relevant data protection regulations, and access to deidentified data was restricted to authorized personnel only. No compensation was provided to participants for their involvement, as the study was conducted as part of standard clinical care during the HAG2022. Furthermore, no identifiable images of individual participants were included in the study, ensuring the confidentiality of all participants in accordance with ethical standards. Patient privacy is ensured and protected through data deidentification techniques.

The research was further approved by the Ethics Committee of Sir Run Run Shaw Hospital, Zhejiang University School of Medicine (Srrsheca2023-0597) and registered at ClinicalTrials.gov (NCT06623825).

## Results

Following the completion of the research period, there were 80 patients categorized into the ARTS cohort (n=10) and the non-ARTS cohort (n=70). Sex and age demographics for the ARTS and non-ARTS cohorts are shown in Table 1. Both cohorts were 70% (ARTS: n=7 and non-ARTS: n=49) male and 30% (ARTS: n=3 and non-ARTS: n=21) female, with no significant gender differences (*P*>.99). The average age for the ARTS cohort was 38.6 (SD 21.0) years and for non-ARTS was 33.3 (SD 12.7) years, with no significant age gap (*P*=.51). Results indicate no significant variations in gender or age between the 2 cohorts.

Table 2 shows a comparison of time intervals for ARTS and non-ARTS cohorts at a tertiary medical center. ARTS group’s average PPT was 23 minutes, significantly lower than the non-ARTS group’s 40.3 minutes (*P*<.001). In the ARTS cohort, the CRT was 15.6 minutes, significantly lower than the non-ARTS cohort’s 164.8 minutes (*P*=.03). The result indicates smart glasses efficiency in reducing PPT and CRT.

Table 3 shows the distribution of patients by diagnosis in the study population. This table provides a detailed breakdown of the number of patients and their corresponding percentages for each diagnosis. Orthopedics or injury emerged as the most frequent diagnosis, with 29 cases, constituting 36.25% (n=26) of the overall study cohort. Following closely was respiratory medicine making up 12.5% (n=10) of the total. This distribution provides insights into the prevalence of different diagnoses within the study population, which can be valuable for understanding the spectrum of conditions during the study (see Table 3)

**Table 1.** Comparison of sex distribution and age demographics between the augmented reality telemedicine system (ARTS) group and the non-ARTS group.

Demographics	ARTS	Non-ARTS	*P* value
Sex, n (%)			>.99
Male	7 (70)	49 (70)	
Female	3 (30)	21 (30)	
Age (years)			.51
Mean (SD)	38.6 (21.0)	33.3 (12.7)	
Median (IQR)	31.0 (16‐81)	30.5 (16‐71)	

**Table 2.** Time intervals statistics by 2 groups: comparison of prepreparation time (PPT) and consult response time (CRT) between the augmented reality telemedicine system (ARTS) group and the non--ARTS group.

	ARTS	Non-ARTS	*P* value
PPT^a^ (min)			<.001
Mean (SD)	23.0 (8.8)	40.3 (22.7)	
Median (IQR)	26 (10‐35)	35 (5‐150)	
CRT^b^ (min)			.03
Mean (SD)	15.6 (4.4)	164.8 (51.8)	
Median (IQR)	14 (10‐25)	162 (71‐307)	

a PPT: prepreparation time from the patient arrival at the door of tertiary hospital to get crucial exams.

b CRT: consult response time from the request to the consultant reaching;

**Table 3.** Distribution of patients by diagnosis for patients admitted to the emergency department (ED) during the 19th Asian Games and Fourth Asian Para Games Hangzhou 2022 (HAG2022).

Diagnosis	Total patients, n (%)	ARTS^a^, n (%)	Non-ARTS, n (%)
Otolaryngology	5 (6.25)	0 (0)	5 (100)
Obstetrics and gynecology	2 (2.5)	1 (50)	1 (50)
Infectious diseases	2 (2.5)	1 (50)	1 (50)
Anorectal surgery	1 (1.25)	0 (0)	1 (100)
Orthopedics or injury	29 (36.25)	5 (17.24)	24 (82.75)
Respiratory medicine	10 (12.5)	1 (10)	9 (90)
Urology	3 (3.75)	1 (33.33)	2 (66.66)
Dermatology	5 (6.25)	0 (0)	5 (100)
General surgery	6 (7.5)	0 (0)	6 (100)
Neurology	2 (2.5)	1 (50)	1 (50)
Gastroenterology	9 (11.25)	0 (0)	9 (100)
Cardiology	4 (5)	0 (0)	4 (100)
Ophthalmology	2 (2.5)	0 (0)	2 (100)

a ARTS: augmented reality telemedicine system.

## Discussion

### Principal Findings and Comparison With Previous Work

This study was conducted at SRRSH in Hangzhou, China, a tertiary referral center affiliated with Zhejiang University School of Medicine. The hospital has around 1200 beds, including an 80-bed Emergency Department that handles an average of 300 patients per day. With its extensive range of consulting specialties, SRRSH played a pivotal role in emergency care, making it a crucial medical hub during the HAG2022. This study highlights the effectiveness of AR smart glasses in improving patient care efficiency and reducing ED wait times, particularly in resource-constrained settings and during mass gatherings like large-scale sports events. The research focused on 2 key time intervals: PPT and CRT. The key findings of this study reveal that AR smart glasses significantly reduced both PPT and CRT compared to conventional telecommunication methods. Findings revealed that the ARTS group had significantly shorter PPT (23 minutes) compared to the non-ARTS group (40.3 minutes). Similarly, CRT was markedly reduced in the ARTS group (15.6 minutes) versus the non-ARTS group (164.8 minutes), as shown in Table 2. These results emphasize the efficiency of the AR telemedicine system in expediting consultations and decision-making. The reductions in both PPT and CRT demonstrate the potential of smart glasses to enhance the effectiveness of EMS. By allowing doctors to visually assess and consult with patients remotely before their arrival, the smart glasses enable immediate guidance and preparation of necessary resources. This real-time interaction ensures that the ED team is well-prepared to deliver prompt care and decisions as soon as the patient arrives, potentially leading to improved clinical outcomes.

Previous studies show that the most common use of smart glasses is in medical education and telementoring, with a growing application in surgical and procedural training, highlighting their potential to enhance hands-on learning and remote guidance in medical settings [23]. Studies show smart glasses in EMS offer significant benefits by enabling hands-free communication, real-time data sharing, and seamless documentation, ultimately improving workflow efficiency and enhancing patient care in high-pressure environments. These devices also assist in secondary triage during mass casualty incidents by transmitting live video feeds to hospital physicians, enabling them to make informed decisions based on real-time data. As a result, the triage accuracy rate increased significantly, from 58% in the conventional method to 92% when using the AR triage algorithm, demonstrating a substantial improvement in the quality of triage [21,24,25]. To the best of our knowledge this study is considered the first study to implement AR glasses in real-case scenarios during a large-scale sport game competition to assess PPT and CRT. The significantly lower PPT and CRT of the ARTS group compared to the non-ARTS highlights the value of AR smart glasses in providing a first-person, immersive view that could further enhance real-time assessments and decision-making processes to support clinical decisions and optimize workflow in the ED (see Figure 2).

### Limitations

**Figure 3.**
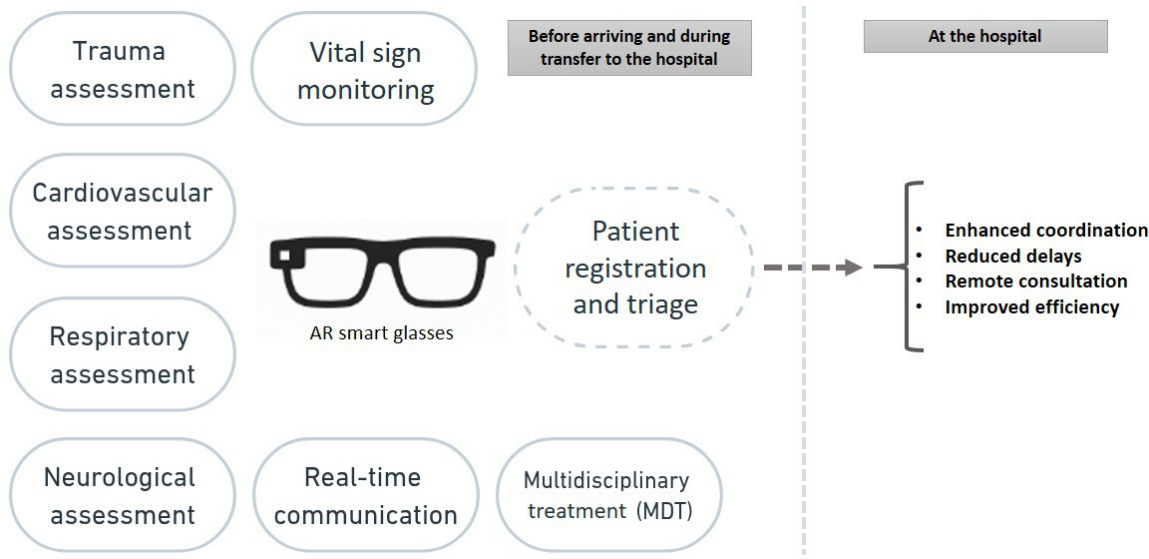
This figure emphasizes the tasks that augmented reality (AR) addresses most effectively. AR enhances even simpler tasks, such as patient registration, by enabling data collection during transportation and minimizing delays.

This study was conducted during the HAG2022, with SRRSH serving as the main tertiary referral center. However, other medical facilities in the region also managed emergency cases, and not all patients from the event were admitted to SRRSH. This study encountered several limitations and challenges including the deployment of AR smart glasses was limited by the availability of the smaller size of the ARTS group (n=10) compared to the non-ARTS group (n=70), which reflects logistical challenges during HAG2022, including single-center nature of the study and the limited availability of AR smart glasses to only 3 units. Therefore, necessitating careful case prioritization and the need for trained personnel to operate them. Despite these constraints, appropriate statistical methods were employed to address potential biases caused by unequal group sizes. A key concern of the study was the potential disruption to regular workflows. To address this, the successful implementation of AR smart glasses was supported by dedicated teams of trained personnel, ensuring that regular ED staff could continue their routine tasks without added strain. However, for broader adoption in typical hospital settings, an assessment of resource requirements, including device availability, staff training, and the impact on existing workflows, would be necessary to ensure successful integration into clinical environments. In addition to tasks that benefit from AR, such as trauma and neurological assessments (eg, stroke evaluations), where visual and real-time interaction is essential, AR will also support the maintenance of a multidisciplinary treatment (MDT), enabling physicians from various specialties and departments to collaborate and manage patient care. There are certain simpler tasks like patient registration and basic triage that can be efficiently handled without the need for AR. Nevertheless, even in these cases, AR contributes value by enabling the collection of patient data during transport, which minimizes delays upon arrival and enhances the ED’s preparedness (see Figure 3). There was 5G technology used in this study; although it enabled minimal latency and high speed however a subset of patients was excluded from the study due to technical difficulties, such as connectivity issues and device malfunctions. Connectivity problems, including network congestion and wireless interference, were especially prominent in the crowded environment of the games and often disrupted real-time communication. Device-specific malfunctions, such as screen freezes, audio failures, and hardware glitches, further hindered the reliability of the AR smart glasses. In some cases, these challenges necessitated the reassignment of AR-assisted cases to the non-ARTS group. While this reassignment caused minimal delays in PPT and CRT, it did reduce the number of cases in the ARTS group, limiting the dataset size. While these challenges were promptly addressed using backup communication systems, Another limitation of this study is the absence of detailed data on the frequency and impact of technical difficulties, with specific data not being collected on how these issues affected patient care, particularly in time-sensitive conditions such as acute coronary syndrome or stroke. The lack of this data limits our understanding of the reliability of AR technology in critical medical scenarios, which is important for future implementations. Although these disruptions resulted in only minimal delays in PPT and CRT, they did impact the statistical power of the study. Furthermore, differences in patient diagnoses, particularly the higher number of orthopedic injuries in the ARTS group may influence the results. These gaps should be addressed in future studies to assess the overall effectiveness of AR in emergency settings.

### Conclusions

In conclusion, this study demonstrates the potential of AR smart glasses in improving the efficiency of emergency care by reducing prepreparation and consultation response times. The findings suggest that AR technologies could be particularly beneficial in emergency departments, especially in high-demand, resource-constrained environments like those experienced during large public events. To improve the implementation of AR smart glasses in emergency medical settings, future studies should focus on addressing the challenges. Enhancements such as improved network infrastructure, more reliable hardware, and comprehensive troubleshooting training for personnel are essential. In addition, future research with larger population and longer periods is needed to validate these findings and explore the scalability of AR telemedicine in diverse emergency scenarios.

## Supplementary material

10.2196/65617 Multimedia Appendix 1 Establish multilevel system of Asian Games medical support based on an augmented reality (AR) real health care system.

## Abbreviations

AR: augmented reality
ARTS: augmented reality telemedicine system
CRT: consult response time
ED: emergency department
EMS: emergency medical services
HAG2022: 19th Asian Games and Fourth Asian Para Games Hangzhou 2022
PPT: prepreparation time
SRRSH: Sir Run Run Shaw Hospital
