# Supplementary material for: Evaluating the Effectiveness of Smart Glasses in Reducing Patient Care Time in Emergency Departments: Cohort Study From the Hangzhou Asian Games
Source: JMIR Form Res. 2025 Jun 30;9:e65617. doi: 10.2196/65617 (PMC12234398; doi:10.2196/65617)
Supplement: Multimedia Appendix 1 [file formative-v9-e65617-s001.pdf]

Establish multi-level System Of Asian Games Medical Support Based On AR RHS

Jiang Xinwei; Xia Bangbo; Hong Yucai
Sir RunRun Shaw Hospital

Issues of emergency in sports fields

Weak pre-hospital first aid ability

The capacity of staff on-site field medical personnel varies greatly

First aid within the fields is separate a nd limited to reach

There are traffic bottlenecks in special cases

How to break through

AR real health system——high quality emergency care

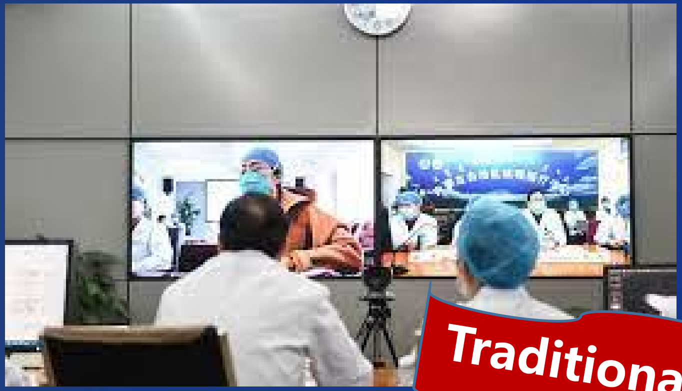

Traditional

Traditional telemedicine

- Fixed place, poor accessibility
- Expensive, difficult to popularize
- Complicated to operate solely
- Only audi-video communication
- Visual rigidity
- Difficult preparation
- Medical records by other hands

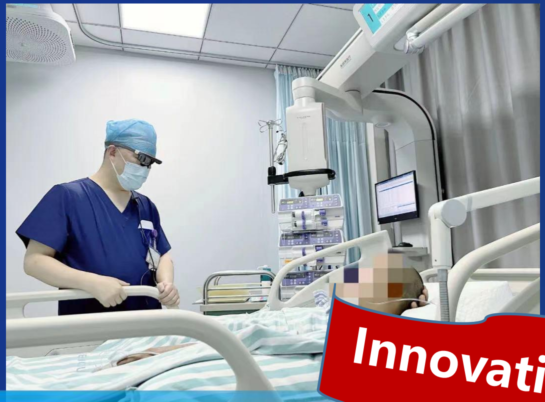

Innovative

AR real health system

- Doctors' operating perspective
- Voice control, hands free
- Handy, accessible for primary hospitals
- AI and self-help auxiliary decision-making
- Anytime and anywhere access to local and national experts
- Audi-video auto-recording

What is AR

- **A**ugmented **R**eality is an interactive experience that combines the real world and computer-generated content.
    - a combination of real and virtual worlds
    - real-time interaction
    - accurate 3-dimensional registration of virtual and real objects

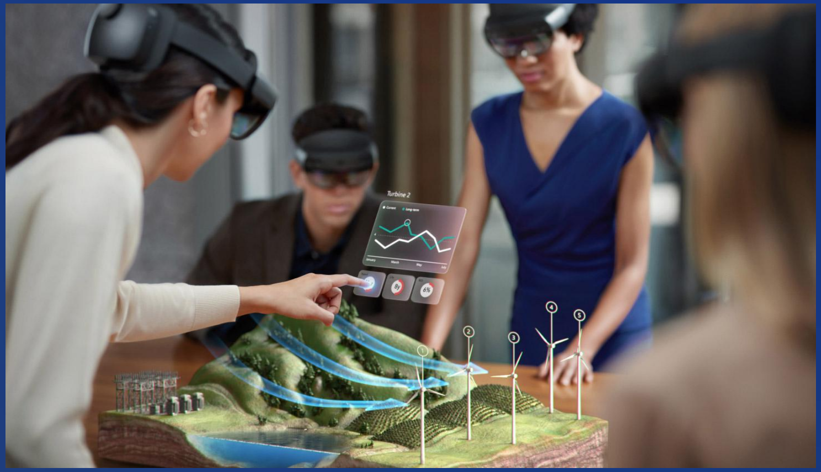

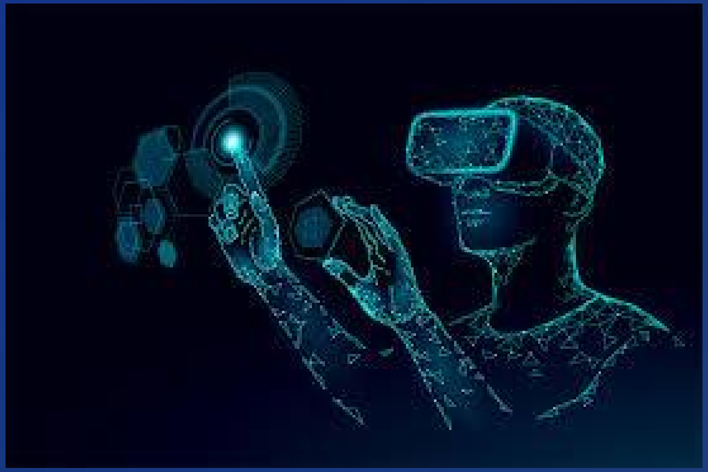

How AR RHS process

- By AR, vital signs & critical information of on-site patients transmitted to experts in real time for remotely assist and realizing the rapid improvement of the emergency medical level of field medical units of Asian Games.

4-5G

On-site rescue **(Main field)**

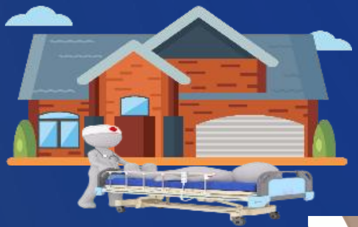

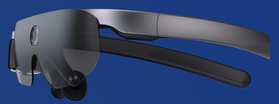

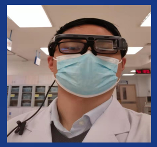

Field doctors wearing AR glasses
receiving assistance of on-site rescue from experts

Intelligent rescue cloud

Ambulance (assistant part)

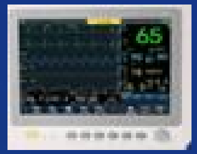

Tele-monitoring

+

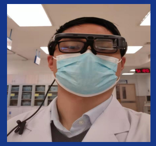

Remote instruction& consultation

Intelligent transport, remote consultation, real-time monitoring on the way.

5G

Tertiary hospital

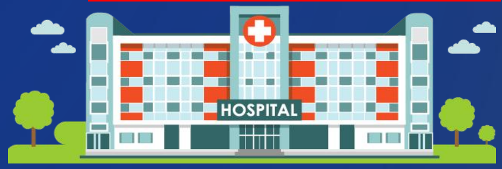

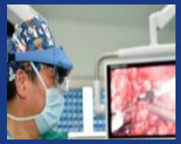

Remote instruction

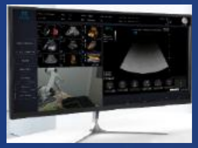

Tele-imaging diagnosis

Remote assistance
Prepare the transfer

Experts remote instruct to backup immediately and entirely

Underlying logic of AR RHS

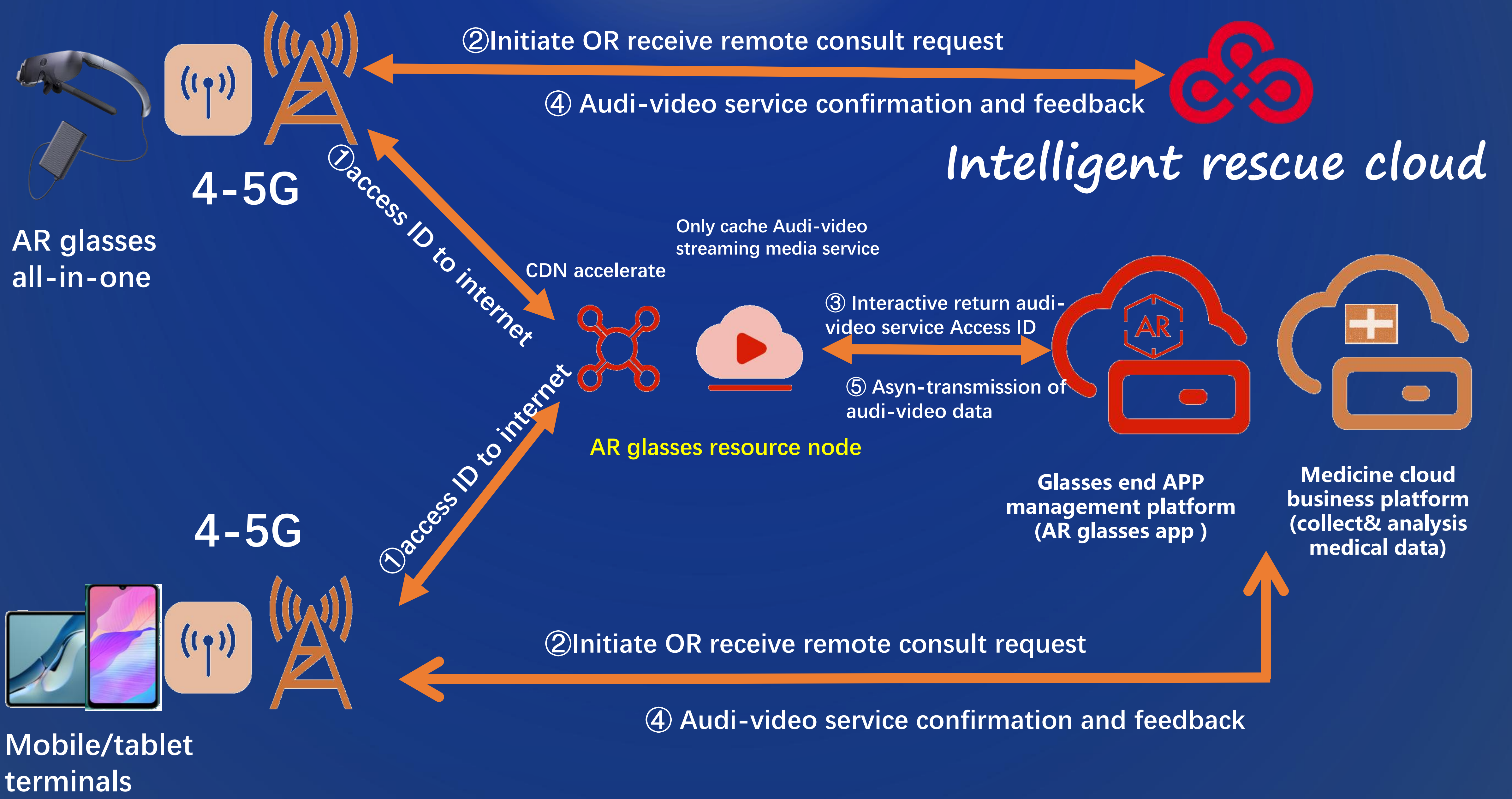

CORE TECHNIQUES

Remote consultation

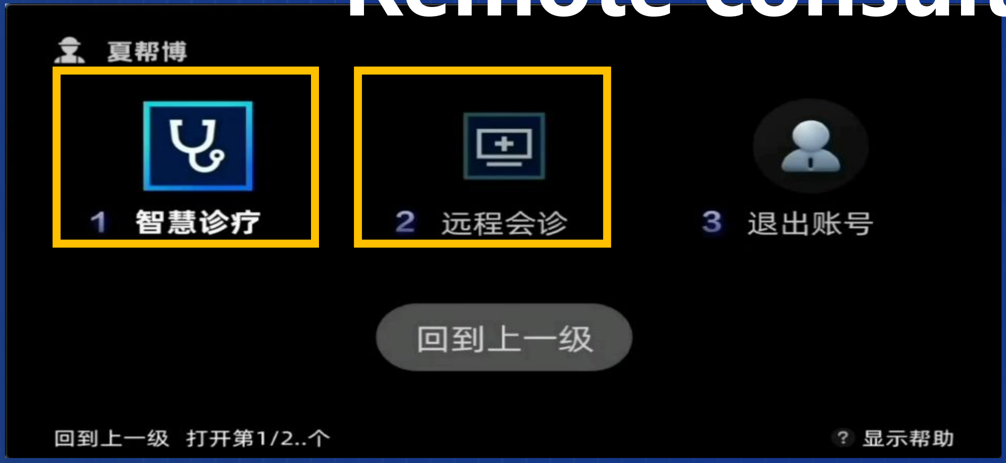

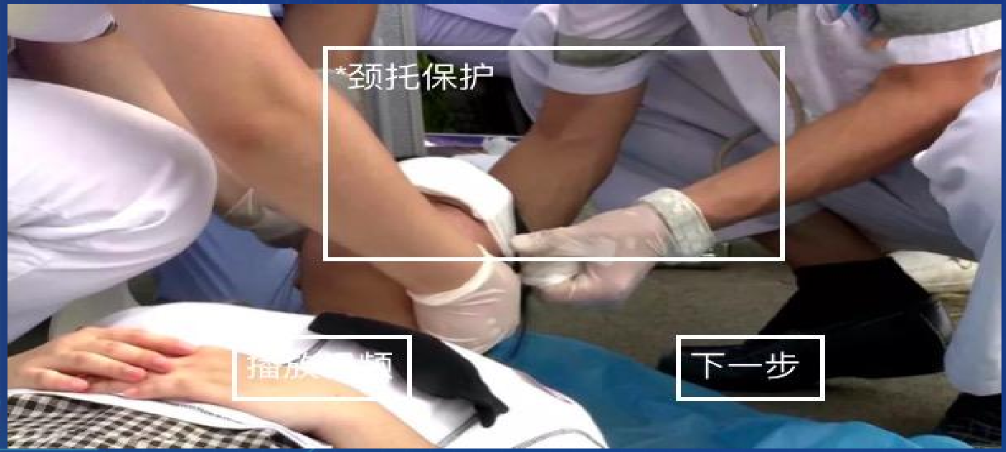

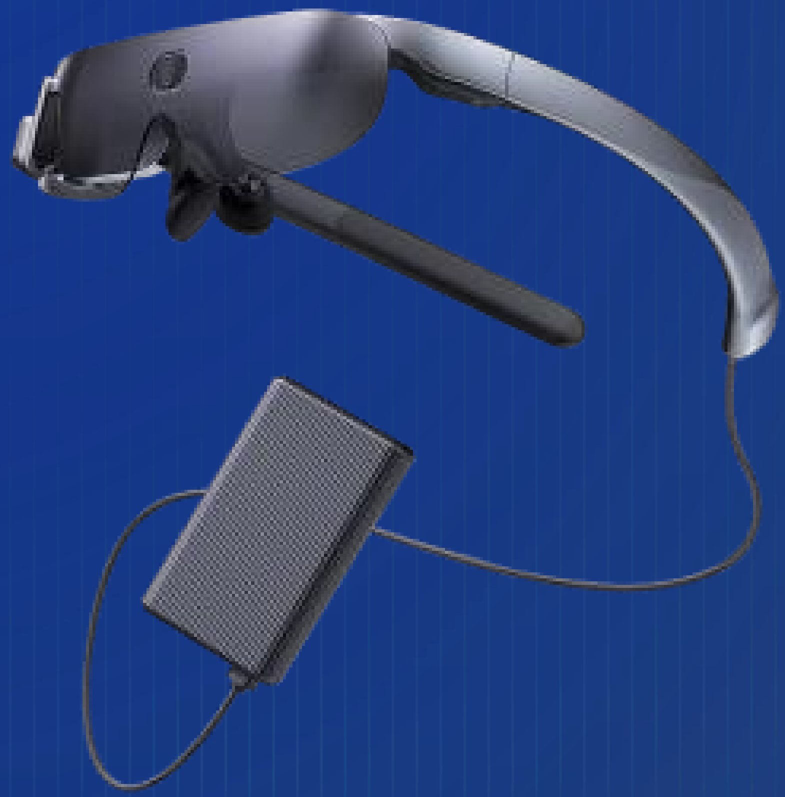

CORE TECHNIQUES

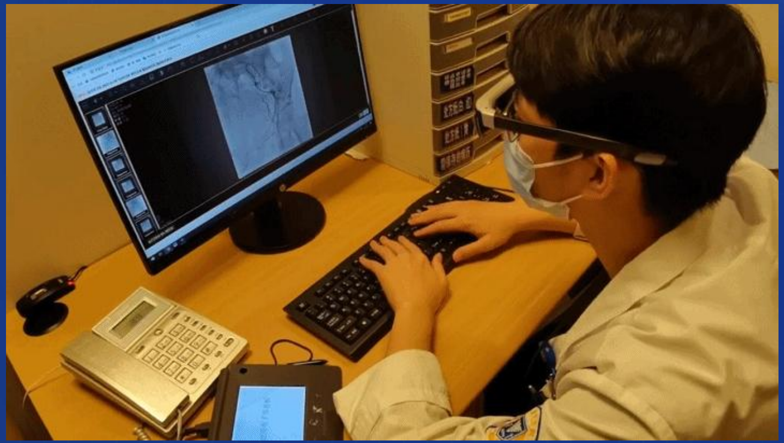

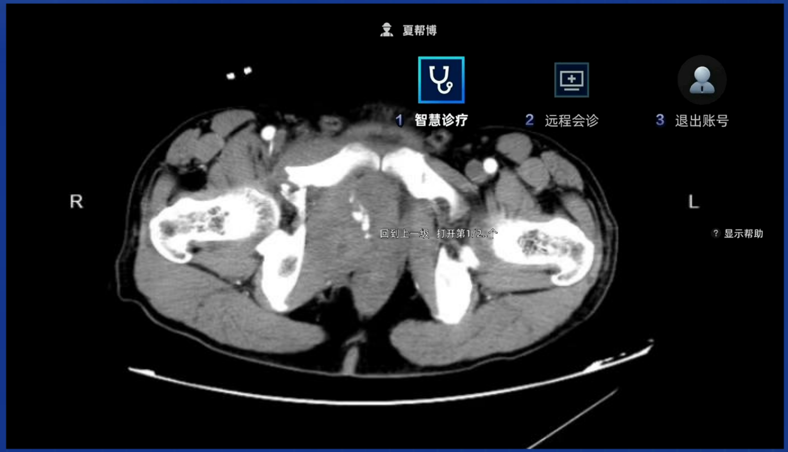

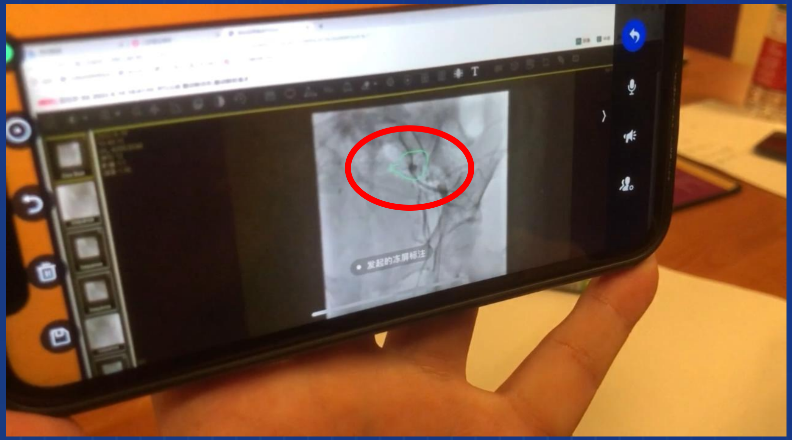

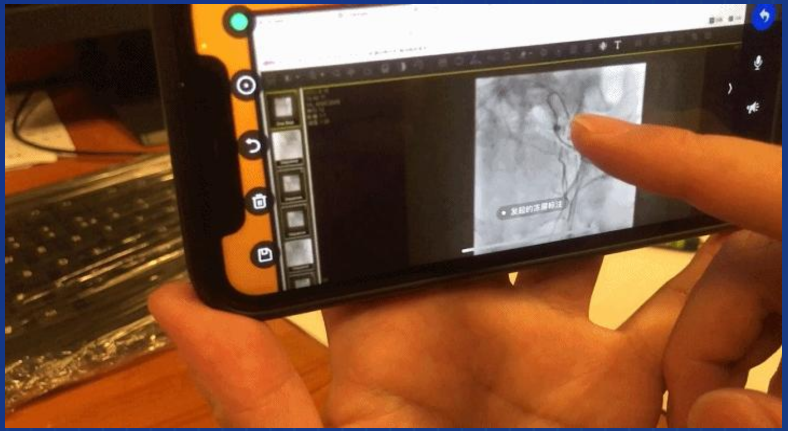

Core function: remote consultation through synchronous transmission

Application scenarios

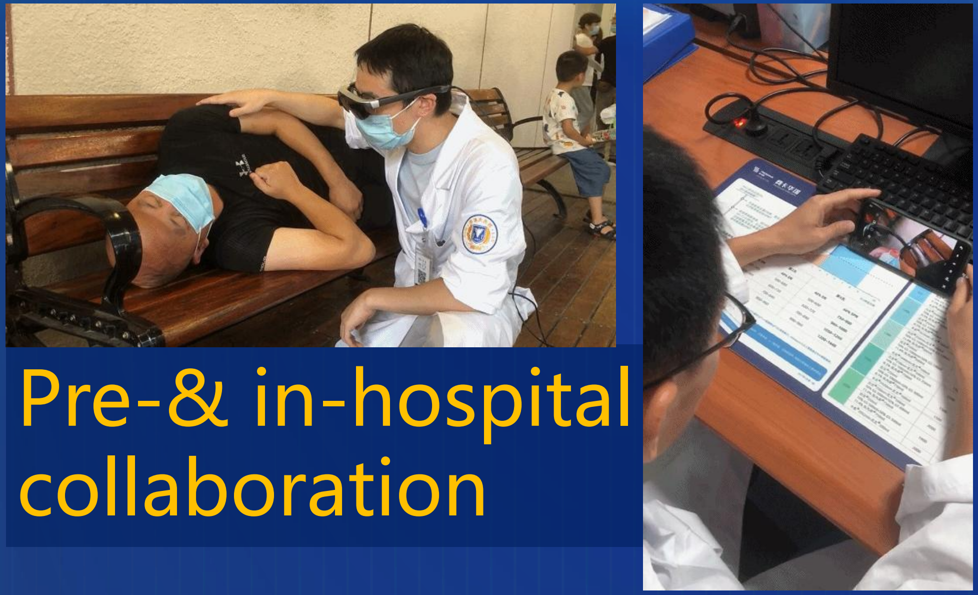

Pre-& in-hospital collaboration

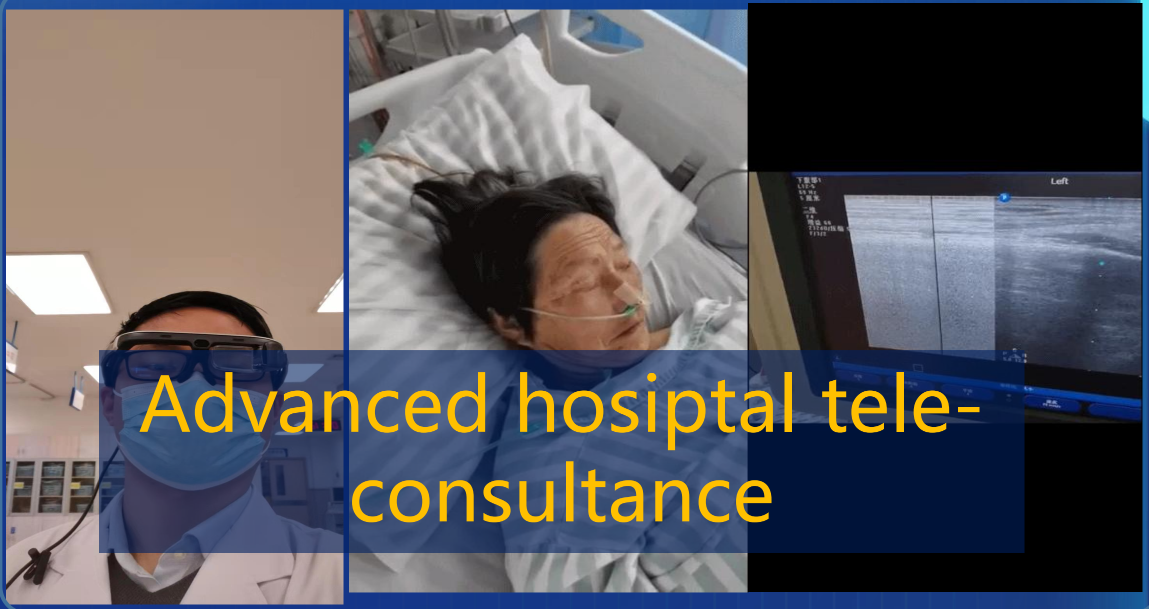

Advanced hospital tele-consultance

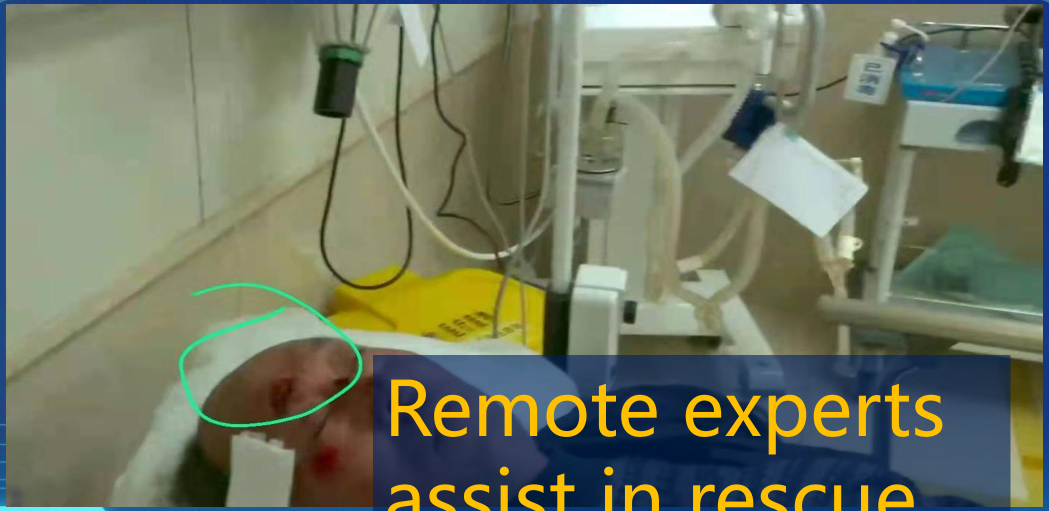

Remote experts assist in rescue

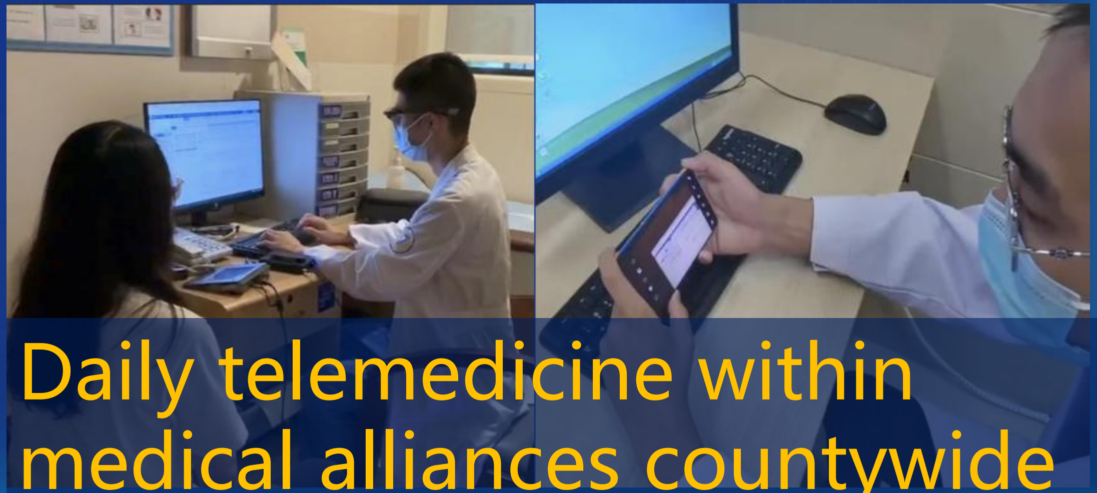

Daily telemedicine within medical alliances countywide

Application case 1

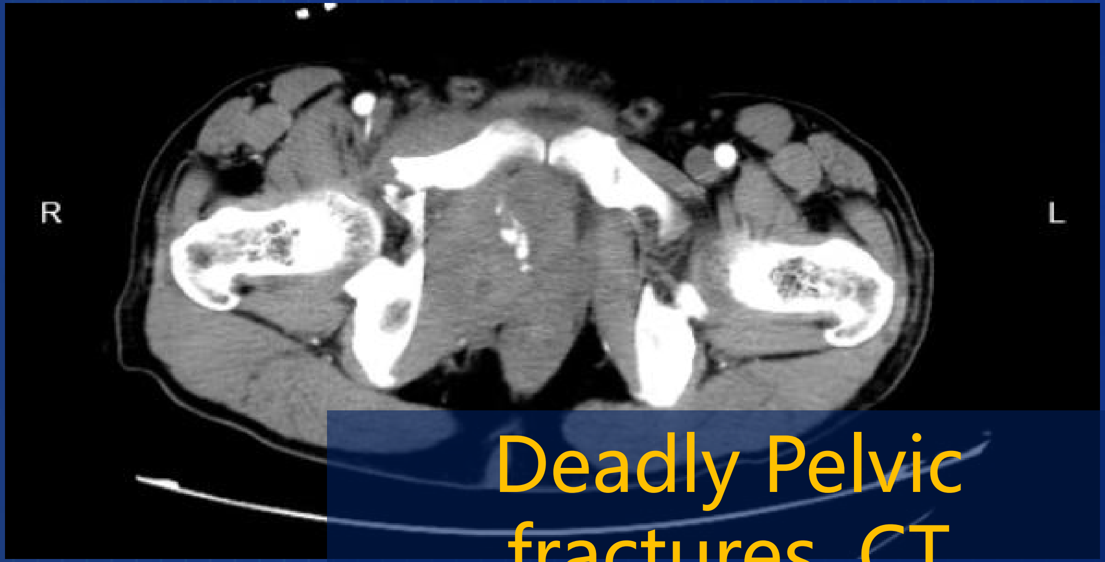

Deadly Pelvic fractures, CT

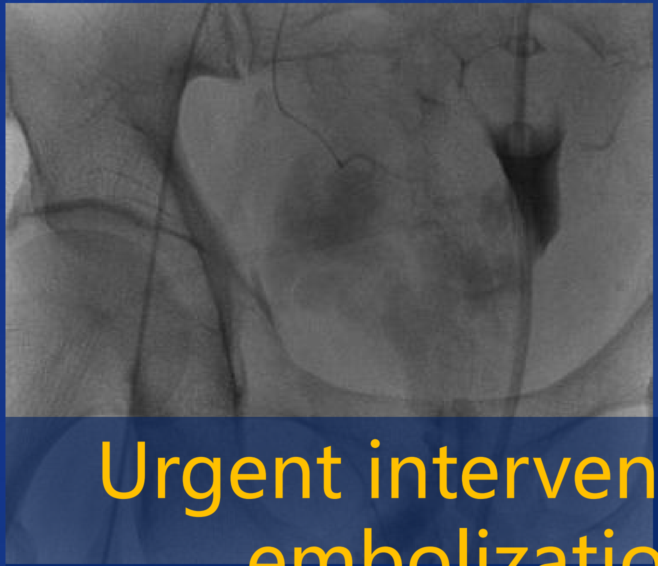

Urgent interventional embolization

Pelvic fracture with massive bleeding in primary hospitals without standardizing pelvic fixation,

DEAD!

Application case 2

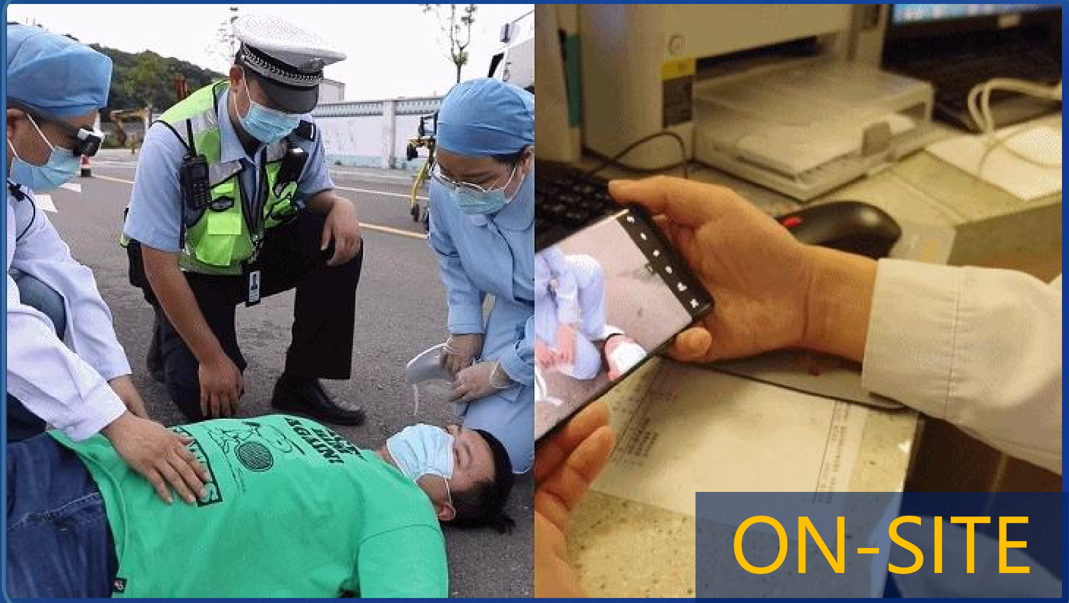

ON-SITE

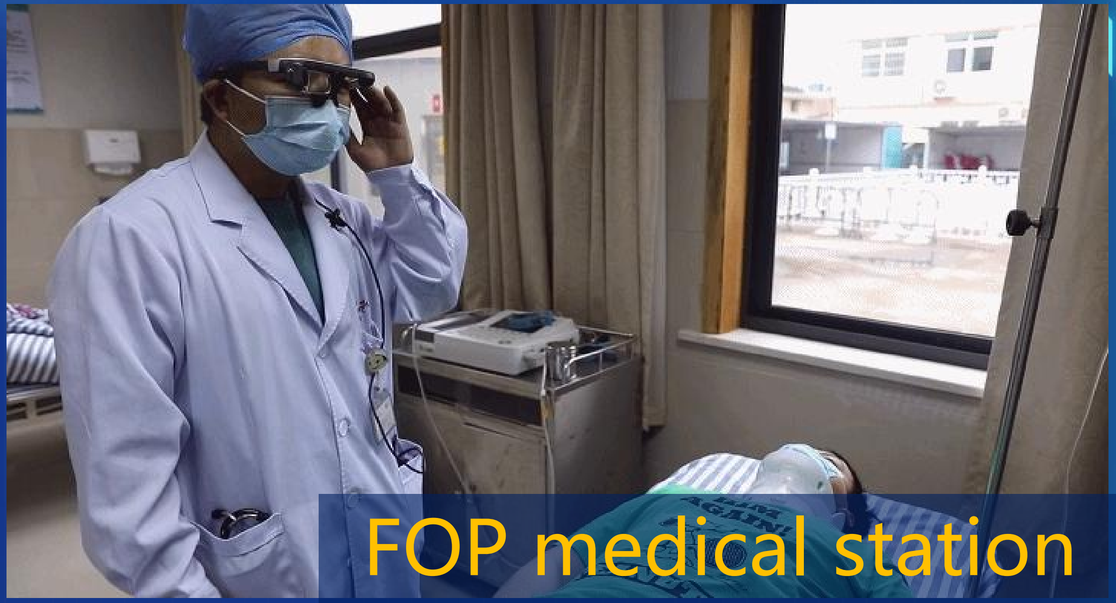

FOP medical station

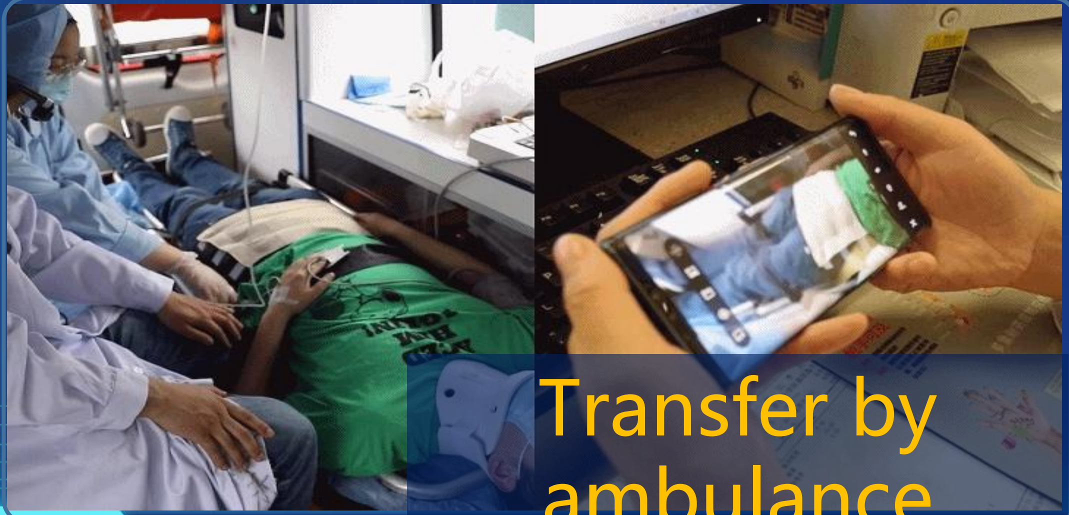

Transfer by ambulance

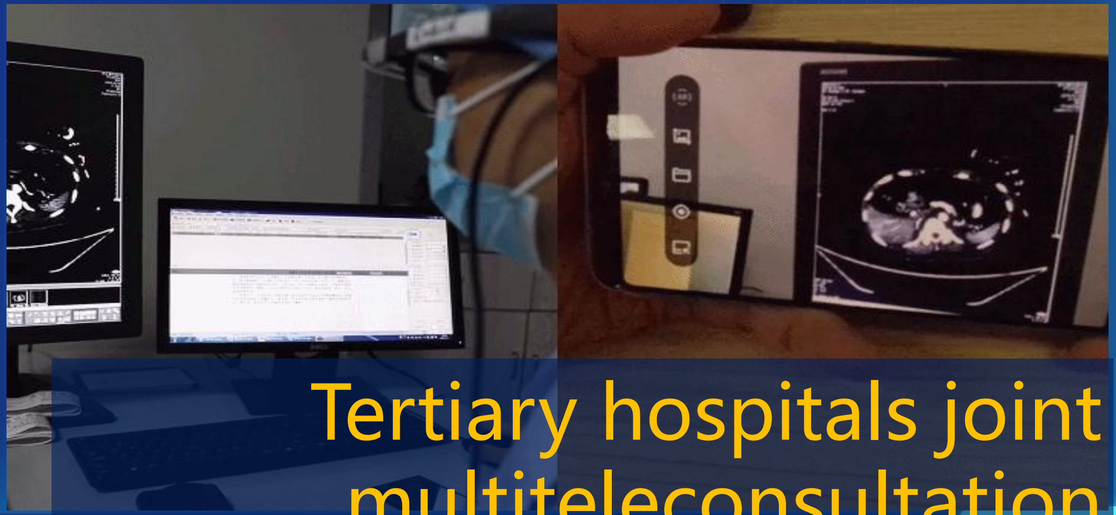

Tertiary hospitals joint multiteleconsultation

Applied regions out of Asian Games

Mountainous area

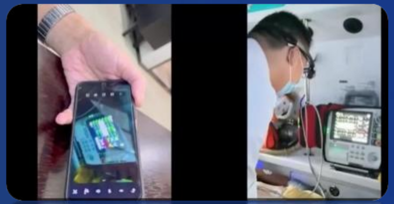

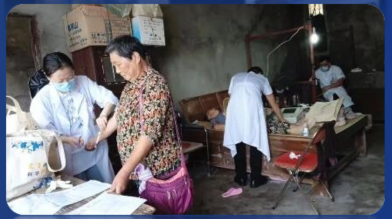

Primary hospitals

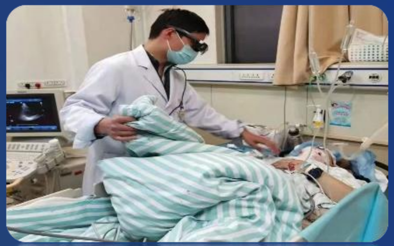

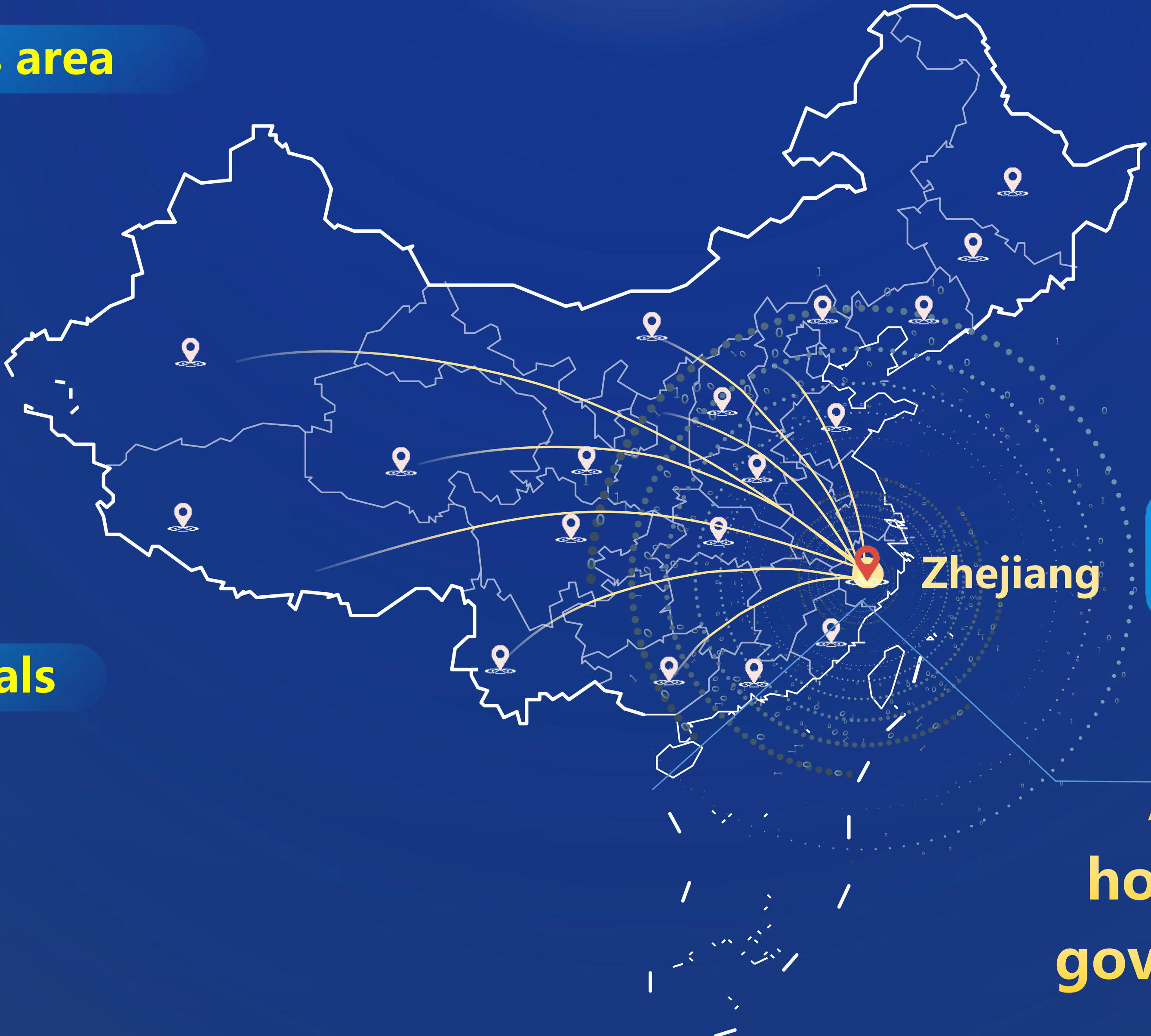

Archipelago

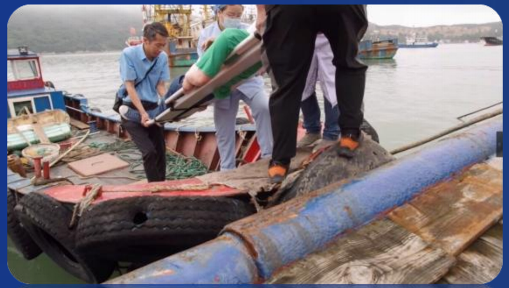

Specific scenes
(prisons,isolation…)

Applied in many hospitals along with governmental support
